# Supplementary material for: Dynamics of trachoma infection in West Africa revealed by a hidden state model
Source: PLoS Comput Biol. 2026 May 20;22(5):e1014313. doi: 10.1371/journal.pcbi.1014313 (PMC13218623; doi:10.1371/journal.pcbi.1014313)
Supplement: S1 Text — Summary of the Markov chain Monte Carlo scheme and hidden infection state sampling (Algorithm S1). (PDF) [file pcbi.1014313.s001.pdf]

S1 Algorithmic description of the Bayesian inference procedure

Algorithm S1 summarises the Markov chain Monte Carlo scheme used to jointly sample model parameters and individual-level hidden infection states. The tuning of this algorithm is described in the main text. The initial state probabilities correspond to the parameters δ^1_{S0}, δ^1_{I0}, δ^1_{ID0}, δ^1_{D0}, δ^1_{S1}, δ^1_{I1}, δ^1_{ID1}, δ^1_{D1}, δ^2_{S0}, δ^2_{I0}, δ^2_{ID0}, δ^2_{D0}, δ^2_{S1}, δ^2_{I1}, δ^2_{ID1}, and δ^2_{D1}. The diagnostic sensitivities and specificities correspond to the parameters ϕ_{PCR}, ψ_{PCR}, ϕ_{ADT}, ψ_{ADT}, ϕ_{CEX}, and ψ_{CEX}. For the mean duration parameters μ_I, μ_{ID0}, μ_{ID1}, μ_{D0}, and μ_{D1}, which are distributed according to a translated negative binomial distribution, we re-parametrise to the probability parameters of the negative binomial distributions, here denoted as ζ_I, ζ_{ID0}, ζ_{ID1}, ζ_{D0}, and ζ_{D1}. These are related according to

$$\mu = 1 + \frac{2(1-\zeta)}{\zeta},$$

where 2 is the size parameter of the negative binomial distribution. When updates are undertaken using random-walk Metropolis proposals, this is shortened to RWM.

The hidden infection states are updated using the individual forward-filter backward-sampler algorithm (IFFBS) [1]. Semi-Markov dynamics arising from negative binomial durations are handled by proposing trajectories from an equivalent Markov model and applying a Metropolis–Hastings correction, ensuring that posterior samples are drawn exactly from the semi-Markov target distribution. The full technical details of this portion of the algorithm can be found in [1].

References

1. Touloupou P, Finkenstädt B, Spencer SEF. Scalable Bayesian inference for coupled hidden Markov and semi-Markov models. Journal of Computational and Graphical Statistics. 2019;29(2):238-49. doi:10.1080/10618600.2019.1654880.

Algorithm S1 Bayesian inference for the individual-level trachoma model

Input: Diagnostic data, household structure, ages, prior distributions
Output: Posterior samples of parameters and hidden infection states

Initialise model parameters
Initialise hidden infection states
for $m = 1$ to M **do** ▷ MCMC iterations
    // Update model parameters given hidden states
    Update unknown ages using independence Metropolis proposals
    Update initial state probabilities via Gibbs sampling
    Update diagnostic sensitivities and specificities via Gibbs sampling
    Update historic infection rate parameter γ^1 using a RWM proposal
    Update historic infection rate parameter γ^2 using a RWM proposal
    Update transmission parameters β_1^1, β_2^1, β_3^1 using RWM proposals in a single block
    Update transmission parameters β_1^2, β_2^2, β_3^2 using RWM proposals in a single block
    Update probability parameter ζ_I using a RWM proposal
    Update probability parameter ζ_{ID0} using a RWM proposal
    Update probability parameter ζ_{ID1} using a RWM proposal
    Update probability parameter ζ_{D0} using a RWM proposal
    Update probability parameter ζ_{D1} using a RWM proposal
    Update reinfection parameter ρ using a RWM proposal
    Update length-biasing reinfection probability parameter α^1 using a RWM proposal
    Update length-biasing reinfection probability parameter α^2 using a RWM proposal
    // Update hidden states given model parameters (IFFBS)
    for each individual $i = 1, \ldots, N$ **do**
        Construct a Markovian proposal model for the hidden states, replacing semi-Markov durations with geometric durations
        Run modified forward filtering algorithm, conditioning on the hidden states of other individuals
        Sample hidden states via backward sampling
        Accept or reject hidden states using MH correction
    end for
    Store posterior samples
end for
return posterior samples
