# Supplementary material for: Dynamics of trachoma infection in West Africa revealed by a hidden state model
Source: PLoS Comput Biol. 2026 May 20;22(5):e1014313. doi: 10.1371/journal.pcbi.1014313 (PMC13218623; doi:10.1371/journal.pcbi.1014313)
Supplement: Attachment — Submitted filename: Trachoma review paper.pdf [file pcbi.1014313.s002.pdf]

The paper presents a complex individual-scale model of trachoma transmission. The authors apply this model to a longitudinal testing dataset across two villages in The Gambia. In the model, individuals move between epidemiological states using both Markovian and non-Markovian dwell time distributions. Inference is performed by approximating the model as a hidden Markov model and performing individual forward-filtering back-sampling.

While the results produced by the model are impressive and likely to have positive public health value, the current statistical methodology section requires further detail before the validity of these results can be fully assessed. In particular:

- Some discussion of what motivated the use of the specific MCMC proposals (lines 171–177) would be helpful. Were these introduced in Touloupou 2017? How were the proposal covariance matrices for the random-walk Metropolis produced from pilot runs?

- The approximation of the semi-Markov model via substitution of the negative binomial distributions with geometric distributions may be valid, but little discussion is made of this point. Is this an approach introduced by Touloupou 2017? Given the negative binomial distributions had size 2, why not capture this exactly by duplicating each state? Given the authors discuss distributions downstream of the infectious period (e.g. secondary case distribution), it seems like it is important that this approximation does not affect results heavily. Ideally, a simulation study could be completed to assess the ability of the model to recover known results (or is Touloupou 2017 sufficient evidence?).

- Further technical detail on the inference approach in general would be appreciated. I examined the code but it is many thousands of lines, so presumably there is some level of detail here that is not reflected in the manuscript. For this and the above points, supplementary materials may be the ideal location to avoid disrupting the flow of the manuscript.

Regarding the results:

- The credible interval for the reinfection parameter rho is very wide yet much discussion is made of its mean being above 1 (line 258). If 41% of the inferred distribution is below 1, this discussion should be further tempered. This section could also potentially be improved by examining if rho has correlations with other parameters.

In addition, I have a number of comments on changes that could be made to improve clarity of the work:

- The specification of the negative binomial distribution (line 122) is ambiguous as there are multiple possible formulations that are commonly used. A specific formula for the pmf would resolve this.

- The language around the distribution tau_I is slightly confusing due to the presence of two distributions: the underlying NB(mu_I - 1, 2) distribution and the translated NB(mu_I - 1, 2) + 1 distribution. I think wording could be changed to make this clearer.

- I was not aware of the term “length-biased” sampling (line 191) prior to reading the manuscript. A short sentence or two explaining the reason why this sampling method produces an upwards bias would be helpful.

- On line 277, it is noted that the size parameter *k* for the distribution of secondary case count indicates lower dispersion than other directly transmitted pathogens. This is an interesting result but would be clearer with some more detail. What are the other pathogens being compared? Is 1 the baseline value of *k*?

- I am confused by the definition of the cumulative probability of infection (line 286). This seems to be the probability of infection under prolonged exposure to the constant probability of infection at time *t*, rather than a cumulative probability (which would need to account for time-varying p_t)? Further, the notation for p_i^t seems to vary between equations.

I also have a few minor comments:

- “unreliable in under one 1’s” (line 77) is a typo

- On line 298, it is noted that children account for a majority of the infection pressure “despite accounting for less than half the population”. However, this is not corroborated by the demographic information provided in Table 1 (with children making up over half the population)

- It seems inappropriate in tone for the authors to conclude that their study sets a gold standard for inferential epidemiology (line 394).
