# Supplementary material for: Dynamics of trachoma infection in West Africa revealed by a hidden state model
Source: PLoS Comput Biol. 2026 May 20;22(5):e1014313. doi: 10.1371/journal.pcbi.1014313 (PMC13218623; doi:10.1371/journal.pcbi.1014313)
Supplement: Attachment — Submitted filename: Response to Reviewers.pdf [file pcbi.1014313.s004.pdf]

Response to Reviewer Comments

April 29, 2026

The comments from the editor and reviewers have been listed in order below, followed by our responses in red text.

Associate Editor

Q 0.1 *Thank you for submitting your manuscript to PLOS Computational Biology. After careful consideration, we feel that it has merit but does not fully meet PLOS Computational Biology's publication criteria as it currently stands. Therefore, we invite you to submit a revised version of the manuscript that addresses the points raised during the review process. The reviewers are incredibly positive about your work, but there are some missing methodological details that are required (see reviewer 3).*

Reply: Thank you for taking the time to consider our manuscript. We have addressed all points raised by the reviewers as summarised below.

Reviewer 1

Q 1.1 *Thank you for this important paper I have minimal suggestions for improvements.*

Reply: Thank you for reviewing our manuscript, below we include answers to specific points.

Q 1.2 *In the section Traversal times you might consider renaming as durations in disease states and provide results as a table with the symbols also explained in words.*

Reply: We have replaced references to traversal times with durations, and presented the results in a table as suggested (Table 4).

Q 1.3 *Results - you present the findings separately for each village which is helpful but I wondered how you might consider combining the data from two or more villages to potentially give more accurate estimates of the key biological parameters?*

Reply: In the manuscript we do undertake a single analysis using data from both villages. To make this clear we have added a few sentences in the inference methodology section (lines 183–188):

"Although some model parameters are village dependent, we fit the model to the data from both villages in a single analysis. Combining data from both villages increases the information available for the shared parameters, directly improving the precision of their estimates. Since the village-dependent parameters may depend on these shared quantities, estimates of these parameters may also benefit indirectly from the joint analysis."

Beyond this, it is not straightforward to aggregate village-dependent parameter estimates into a single generalised value. For example, transmission parameters vary with local factors such as age structure,

household density, and geography. For this reason, we focus on joint inference of shared parameters, while allowing village-specific parameters to capture genuine contextual differences.

Q 1.4 *Discussion highlights clearly the implications of the findings. R0 remains >2 which presents significant challenges for control when reinfection is common. Are there other harm reduction methods alongside vaccines e.g. related to WASH or other specific practices within the home?*

Reply: We have expanded the discussion to address other harm-reduction approaches alongside vaccines, particularly WASH. There is mixed evidence for the impact of WASH on trachoma prevalence from observational studies and randomised trials. The new paragraph is as follows (lines 443–457):

"Our results suggest that transmission-blocking interventions targeted at young children could substantially reduce trachoma transmission within communities. Current elimination programmes are largely based on the SAFE strategy (surgery for trichiasis, antibiotics, facial cleanliness, and environmental improvement), which encompasses WASH (water, sanitation, and hygiene) [8, 34]. Annual mass treatment with azithromycin remains the cornerstone of control efforts, and has proven effective at reducing trachoma infection and the resulting blindness [3, 6]. While observational studies have reported associations between household water access or facial cleanliness and lower odds of active trachoma [35, 36], randomised trials in Ethiopia have not demonstrated a measurable impact of WASH provision on trachoma prevalence [10, 37]. Despite major progress towards elimination, persistent endemic foci remain, most notably in Ethiopia, prompting renewed interest in novel interventions such as vaccines against Chlamydia trachomatis [38]. A candidate antigen, CTH522, is currently in clinical trials [39], and our findings add to the evidence base supporting the prioritisation of transmission-blocking vaccines targeted at children."

Q 1.5 *I was able to locate the code and supplementary files at zenodo although I have not reviewed these in detail. Overall I found this paper super interesting and a great application of the methods to this ongoing challenge. I look forward to seeing the method applied to other infections to combined multiple data sources.*

Reply: Thank you very much for the kind words, we are pleased to hear you enjoyed our manuscript!

Reviewer 2

Q 2.1 *This paper presents a very nice re-analysis of unique data, and offers a variety of intriguing outputs that are unlikely to be estimated based on empirical data in any other way. For that reason it's a technically significant manuscript. The authorship team is exemplary. My comments are all very pedantic.*

Reply: Thank you for reviewing our manuscript, below we include answers to specific points.

Q 2.2 *Line 11: rather than "currently" suggest specify a date – this paper will be read and used for a while to come.*

Reply: We have added March 2026 as the date, updated the text to reflect the latest information, and updated the date in the reference accordingly.

Q 2.3 *Line 16: please use an em-dash between "inflammation" and "follicular", as specified in the report of the 4th Global Scientific Meeting on Trachoma. You've written it correctly in line 86, but at that point the abbreviation has already been defined.*

Reply: We now use an em-dash, and have removed the repeated definition.

Q 2.4 *Line 20: please change "face washing" to "facial cleanliness".*

Reply: This has been changed.

Q 2.5 *Line 24: it would be preferable to refer to programmes as "trachoma elimination programmes" – this speaks to the agreed global public health target.*

Reply: This has been changed. We have also changed several instances of 'programs' to 'programmes' for consistency.

Q 2.6 *Line 62 (and subsequently): I don't think "plasmid PCR" is quite right. "PCR targeting a plasmid sequence" is probably ok. Similarly, I've not previously referred to assays as "antigen trap test"; "antigen detection test" would be better.*

Reply: These changes have been made throughout the manuscript.

Q 2.7 *Line 77: please edit "one 1's"; if you retain the digit rather than the word, it doesn't need an apostrophe.*

Reply: This has been corrected.

Q 2.8 *Line 158: TF means a very specific thing. Please don't say "follicular disease (TF)": not all follicular conjunctivitis meets the criteria for TF. Here TF is intended, so just say "TF". Line 235 and 243: please change "follicular trachoma" and "ocular disease (TF)" here to "TF"*

Reply: These changes have been made.

Q 2.9 *Caption Figure 3: Please change "Prevalence of infection with Chlamydia trachomatis (I) and ocular disease with follicular trachoma" to "Prevalence of conjunctival Chlamydia trachomatis infection and trachomatous inflammation—follicular (TF)". Please alter the inset box in the figure area to change "diseased" to "TF" and "Infectious" to "Infected": there is other disease from trachoma other than TF, and "infected" and "infectious" are not necessarily the same thing.*

Reply: These changes have been made.

Q 2.10 *Caption Table 3: please change "becoming infected with trachoma" to "acquiring conjunctival Chlamydial trachomatis infection".*

Reply: This change has been made.

Q 2.11 *Line 259: abbreviation already defined.*

Reply: This has been replaced with 'TF'.

Q 2.12 *Line 268: here I think it's important to qualify that the estimate is of the R0 for "conjunctival" C. trachomatis.*

Reply: This is now qualified.

Q 2.13 *Figure 5 caption: please check that "infectiousness" is really what is being represented here.*

Reply: We have removed the term "infectiousness", as the figure corresponds to susceptibility.

Q 2.14 *Line 296: should 0–9 years be 1–9 years? See Figure 6.*

Reply: We exclude data from under 1s, as clinical signs of trachoma are considered unreliable. However, we still include under 1s in the model, and so they can contribute to the force of infection. We have extended the explanation when describing the data (lines 77–80):

"Note that we exclude data from individuals under 1 year of age as clinical signs of trachoma are considered unreliable in under 1s. These individuals are still included in the model described in the next section, but are treated as having missing data."

Q 2.15 *Suggest add a limitations para to the discussion. This should include the fact that actual transmission events were not observed; nor were they inferable by, for example, sequencing isolates to derived circumstantial evidence of transmission between people sharing rooms.*

Reply: We have added the following paragraph to the discussion (lines 458–469):

"This study also has several important limitations. Transmission events are not observed directly, but are instead inferred from longitudinal diagnostic and clinical observations by fitting a stochastic transmission model. While the availability of repeated measurements alongside the detailed household structure of the population allows us to infer transmission dynamics with greater clarity than cross-sectional data, we still can not unambiguously identify who-infected-whom. Furthermore, we lack complementary data such as pathogen genome sequences that could provide circumstantial evidence of direct transmission between individuals. The transmission parameters therefore represent average effects at the level of shared environments, rather than confirmed person-to-person transmission events. Future studies combining dense longitudinal data with pathogen genomic data could help resolve transmission links more precisely, and further validate model-based inference."

Reviewer 3

Q 3.1 *The paper presents a complex individual-scale model of trachoma transmission. The authors apply this model to a longitudinal testing dataset across two villages in The Gambia. In the model, individuals move between epidemiological states using both Markovian and non-Markovian dwell time distributions. Inference is performed by approximating the model as a hidden Markov model and performing individual forward-filtering back-sampling.*

While the results produced by the model are impressive and likely to have positive public health value, the current statistical methodology section requires further detail before the validity of these results can be fully assessed.

Reply: Thank you for reviewing our manuscript, below we include answers to specific points.

Q 3.2 *Some discussion of what motivated the use of the specific MCMC proposals (lines 171–177) would be helpful. Were these introduced in Touloupou 2017? How were the proposal covariance matrices for the random-walk Metropolis produced from pilot runs?*

Reply: We have expanded this paragraph to include more information about motivation and the use of pilot runs (lines 189–209):

"When updating the model parameters conditional on the hidden infection states we use a variety of MCMC updates that utilise available conjugacy and provide efficient mixing. For the initial state probabilities, sensitivity, and specificity parameters, our choice of conjugate prior distributions means that the full conditional distributions are available, allowing efficient Gibbs updates. For the unknown age parameters we use independence Metropolis proposals using their prior distributions. We find that there is little information in the data to estimate these ages, meaning that the posterior distributions closely resemble the prior distributions, and so this is a more efficient approach than using random-walk proposals. For the remaining parameters, the full conditional distributions are not analytically available, and so we use random-walk Metropolis updates. Transmission parameters for each village are updated jointly in blocks to reduce computational cost. As the three transmission parameters contribute to the infection probabilities, which are needed to compute the likelihood for these updates, blocking these parameters avoids multiple calculations of each infection probability. The mean durations are re-parametrised to the probability parameters of the negative binomial distributions to improve mixing. The random-walk proposal distributions are constructed using empirical covariance estimates obtained from pilot runs. The proposal covariance matrix for each block of transmission parameters is taken to be the empirical covariance matrix scaled by a factor of $2.38^2/3$, following [27].

Likewise, for the remaining parameters the proposal variance is scaled by 2.38^2, as these are univariate proposals."

Q 3.3 *The approximation of the semi-Markov model via substitution of the negative binomial distributions with geometric distributions may be valid, but little discussion is made of this point. Is this an approach introduced by Touloupou 2017? Given the negative binomial distributions had size 2, why not capture this exactly by duplicating each state? Given the authors discuss distributions downstream of the infectious period (e.g. secondary case distribution), it seems like it is important that this approximation does not affect results heavily. Ideally, a simulation study could be completed to assess the ability of the model to recover known results (or is Touloupou 2017 sufficient evidence?).*

Reply: The approximate Markov model is used only to construct efficient proposals via IFFBS. Inference is performed under the semi-Markov model, and the Metropolis–Hastings correction ensures that accepted hidden infection states are distributed according to the exact semi-Markov posterior. Thus, the approach does not approximate the model, but rather uses an auxiliary Markov process to improve computational efficiency. This approach was compared with others in Touloupou 2019, and shown to be highly efficient. This has now been made clearer (lines 215–226):

"As negative binomial durations yield a semi-Markov model, this cannot be done directly. Instead, we first construct a Markovian approximation to our stochastic transmission model by setting the size parameters of the negative binomial distributions to 1 (corresponding to geometric distributions) while maintaining the same mean durations. For each individual, we propose a set of hidden infection states using IFFBS with the approximate model, and then apply a Metropolis-Hastings accept-reject step to correct for the discrepancy between the approximate and correct models. This accept-reject step ensures that the resulting Markov chain has the correct stationary distribution, and posterior samples are drawn from the true semi-Markov model rather than from the Markov approximation. As demonstrated in [21], this is a highly efficient way to sample the hidden states of semi-Markov models, particularly when the size parameters are close to 1, as is the case here."

Regarding replacing the negative binomial distributions with two geometric distributions, we have included the following sentences as an explanation when we first introduce the negative binomial distribution (lines 126–130):

"An equivalent formulation would be to duplicate state I and model transitions through two sequential infectious compartments with geometric durations, yielding a Markov structure [20]. However, because the inference algorithm we employ scales quadratically with the number of model states [21], this formulation would substantially increase computational cost. We therefore adopt the negative binomial specification..."

Q 3.4 *Further technical detail on the inference approach in general would be appreciated. I examined the code but it is many thousands of lines, so presumably there is some level of detail here that is not reflected in the manuscript. For this and the above points, supplementary materials may be the ideal location to avoid disrupting the flow of the manuscript.*

Reply: We have added a dedicated algorithmic summary of the inference procedure to the Supplementary Information (S1 Text). This pseudo-code provides a step-by-step overview of the MCMC scheme, including parameter updates and the hidden state sampling procedure.

Q 3.5 *The credible interval for the reinfection parameter rho is very wide yet much discussion is made of its mean being above 1 (line 258). If 41% of the inferred distribution is below 1, this discussion should be further tempered. This section could also potentially be improved by examining if rho has correlations with other parameters.*

Reply: We have rewritten the paragraph as follows (lines 304–318):

"We assess whether individuals who have previously been infected and have ocular disease (D) are more likely to become reinfected than susceptible individuals (S). The posterior distribution of the reinfection

parameter ρ is broad, with a mean of 1.24 (95% CrI 0.27, 2.79), though the corresponding effect on infection risk is non-linear due to the exponential function (Eq 5). While the posterior mean exceeds 1, suggesting higher susceptibility among individuals with TF in contrast to earlier analyses [29], this estimate is highly uncertain. Notably, 41% of the posterior mass lies below 1, indicating substantial support for the alternative possibility that individuals in the D state are less susceptible than those in the S state. We observe no strong posterior correlation between ρ and other model parameters, indicating that this uncertainty is not driven by parameter confounding but rather by limited information in the data. Although there are plausible biological mechanisms that could increase infection risk among diseased individuals (e.g. increased eye touching or eyelid damage), the data do not strongly constrain ρ, and conclusions regarding differences in susceptibility should therefore be interpreted with caution."

Q 3.6 *The specification of the negative binomial distribution (line 122) is ambiguous as there are multiple possible formulations that are commonly used. A specific formula for the pmf would resolve this.*

Reply: We have added the relevant PMF (lines 130–132 and equation 3):

"We therefore adopt the negative binomial specification, where $\mathrm{NB}(m, n)$ is defined by the following probability mass function:

$$p(x \mid m, n) = \frac{\Gamma(x+n)}{\Gamma(n)x!}\left(\frac{n}{n+m}\right)^{n}\left(\frac{m}{n+m}\right)^{x}, \quad x = 0, 1, 2, \ldots,$$

where m is the mean and n is the size parameter of the negative binomial distribution."

Q 3.7 *The language around the distribution τ_I is slightly confusing due to the presence of two distributions: the underlying $NB(mu_I - 1, 2)$ distribution and the translated $NB(mu_I - 1, 2) + 1$ distribution. I think wording could be changed to make this clearer.*

Reply: We have reworded this section for clarity, particularly stressing when we are discussing the mean of the duration vs. the mean of the negative binomial distribution. For example, when introducing the duration of state I (lines 120–123 and equation 2):

"For individuals in state I we define the duration spent in this state before progressing to state ID. We denote the duration as τ_I and the mean duration as μ_I. Then τ_I is distributed according to a translated negative binomial distribution with mean $\mu_I - 1$ and size 2:

$$\tau_I \sim 1 + \mathrm{NB}(\mu_I - 1, 2)."$$

Q 3.8 *I was not aware of the term "length-biased" sampling (line 191) prior to reading the manuscript. A short sentence or two explaining the reason why this sampling method produces an upwards bias would be helpful.*

Reply: We have added the following few lines as well as a reference (lines 229–235):

"These durations will be upwardly biased due to length-biased sampling [28]. This occurs when the probability that an observation is included in a sample increases with the length (or duration) of the phenomenon being measured. In our setting, the longer the duration of infection, the more likely it is to overlap with the start of the survey. As a result, longer infection durations are overrepresented, and the sample is not representative of the underlying population."

Q 3.9 *On line 277, it is noted that the size parameter k for the distribution of secondary case count indicates lower dispersion than other directly transmitted pathogens. This is an interesting result but would be clearer with some more detail. What are the other pathogens being compared? Is 1 the baseline value of k?*

Reply: We have expanded this section to include the suggested information (lines 328–337):

"The simulated distribution of secondary cases is shown in Fig 4. Fitting the negative binomial distribution to the secondary cases we obtain a value for the size parameter k as 1.81 for village J and 1.61 for village B. A small value of k (e.g. < 1) indicates highly heterogenous transmission, with most secondary cases arising from a small number of individuals ("superspreaders"). As k increases, transmission becomes more evenly distributed across individuals, and the secondary case distribution converges towards a Poisson distribution. A previous study estimated the values of k for several past outbreaks (including SARS, measles, monkeypox, and pneumonic plague) to be in the range of $0.01 - 1$ [30]. Our results therefore indicate a lower dispersion of secondary cases compared with other directly transmitted pathogens."

Q 3.10 *I am confused by the definition of the cumulative probability of infection (line 286). This seems to be the probability of infection under prolonged exposure to the constant probability of infection at time t, rather than a cumulative probability (which would need to account for time-varying p_t)? Further, the notation for p_i^t seems to vary between equations.*

Reply: Thanks for pointing this out. We have amended the definition of the cumulative probability of infection to $Cp_t^i = 1 - \prod_{k=1}^{t}(1 - p_k^i)$ (line 344). The notation p_t^i is now consistent with earlier equations.

Q 3.11 *"unreliable in under one 1's" (line 77) is a typo.*

Reply: This has been corrected.

Q 3.12 *On line 298, it is noted that children account for a majority of the infection pressure "despite accounting for less than half the population". However, this is not corroborated by the demographic information provided in Table 1 (with children making up over half the population).*

Reply: We have clarified that we are referring to young children ($0 - 9$ year-olds) in this section, and the specific percentages have been added for comparison (lines 354–361):

"We examine the relative contribution of different age groups to the weekly probability of trachoma transmission, given by the force of infection (Eq 1). We find that young children (0–9 years) contribute disproportionately to transmission, accounting for more than 75% of the infection pressure in the largest village (J) and more than 70% in the second village (B) over the observed period, despite accounting for less than half of the population (J: 36%, B: 40%), see Fig 6. Adults 16+ years of age contribute under 20% to the force of infection in both villages, which is a consequence of having a lower duration of infectiousness due to past infections."

Q 3.13 *It seems inappropriate in tone for the authors to conclude that their study sets a gold standard for inferential epidemiology (line 394).*

Reply: We have rewritten the final paragraph (lines 470–473):

"Overall, this study demonstrates how a principled statistical approach applied to detailed longitudinal data can be used to jointly infer hidden infection states and key natural history parameters, yielding new insights into the underlying dynamics of trachoma infection."

Additional changes not requested by reviewers

Q 3.14 *During revision, we made a small clarification to the description of prior distributions for diagnostic sensitivities and specificities in the prior distributions section. Specifically, we clarified the rationale for using the same prior distributions for both the PCR and antigen detection tests. This change was made to improve clarity and does not affect the results or conclusions. The full paragraph now reads as follows (lines 248–255):*

Reply: "We use informative prior distributions for the observation sensitivities and specificities based on previous studies (Table 2). Although informative prior distributions can be constructed for the PCR tests from previous studies, comparable data are not available for antigen detection tests in this setting, and we therefore use the same prior distributions for these two diagnostics. This choice reflects prior uncertainty about the diagnostic performance in this setting, while avoiding favouring one test over another *a priori*. For the remaining model parameters, we use weakly informative prior distributions to constrain the parameters within sensible ranges."
